# Incongruent symptom perceptions and its associations with negative mood, intimate relationship and quality of life in colorectal cancer dyads: a dyadic analysis

**DOI:** 10.1186/s41687-026-01090-5

**Published:** 2026-06-06

**Authors:** Xia Wu, Roger W. Chan, Hongge Tang, Yiming Li

**Affiliations:** 1 Department of Nursing, Xiamen Medical College, 1999 Guankou Middle Road, Jimei District, Xiamen, Fujian China; 2 Department of Public Health and Medical Technology, Xiamen Medical College, Xiamen, Fujian China

**Keywords:** Colorectal cancer, Sleep disturbance, Negative mood, Intimate relationship, Quality of life

## Abstract

**Purpose:**

The primary objective of this study was to explore the existence and identify predictors of incongruent perceptions of pain, fatigue, and sleep disturbance, and to examine their associations with negative mood, intimate relationship, and quality of life in colorectal cancer patient- caregiver dyads.

**Methods:**

271 colorectal cancer dyads completed questionnaires designed to assess a range of outcomes, including patient symptoms (pain, fatigue, sleep disturbance), negative mood, intimate relationship, and quality of life. Pearson’s correlation, multiple linear regression analysis and structural equation modeling were used to analyze the data.

**Results:**

There were significant disparities in incongruent perceptions of sleep disturbance between patients and caregivers (*t*=-3.241, *p* < 0.001). For incongruent sleep disturbance, regression analysis showed patient education level (B=-1.014, *p* = 0.001) and caregiver education level (B=-1.140, *p* < 0.001) demonstrated significant negative associations. Longer patient diagnosis duration (B=-1.876, *p* = 0.010) was negatively associated. Caregiver religious affiliation (B=-1.611, *p* = 0.027) and marital status (B= -3.097, *p* = 0.001) showed negative associations. Patient gender (B= 1.361, *p* = 0.037) and caregiver gender (B = 1.803, *p* = 0.004) exhibited positive associations. Dyadic relationship type (B = 1.568, *p* = 0.043) was positively associated. The analysis revealed that incongruent perceptions of sleep disturbance exhibited a significant correlation with the quality of life and negative mood of both individuals within colorectal cancer patient-caregiver dyads. The model fit estimates were as follows: χ2/df = 1.570, GFI = 0.967, CFI = 0.957, IFI = 0.922, RMSEA = 0.036, SRMR = 0.053. Pathway analysis demonstrated that incongruent perceptions of sleep disturbance exerted a significantly direct effect on the quality of life of both individuals in colorectal cancer patient-caregiver dyads, albeit in opposite directions (B=-0.238, *p* < 0.001; B = 0.358, *p* < 0.001, respectively). Furthermore, the analysis revealed an actor-partner mediating effect of incongruent perceptions of sleep disturbance, negative mood, intimate relationship, and quality of life.

**Conclusion:**

The findings of the present study indicated that incongruent perceptions of sleep disturbance were associated with negative mood, low intimate relationship satisfaction, and low quality of life. The individual attributes of patients (gender, educational level and time since diagnosis) and caregivers (gender, educational level, religious beliefs and marital status), as well as dyadic factors such as relationship, could predict patient-caregiver concordance on assessing patients’ symptoms. It is recommended that healthcare providers should reinforce patient-caregiver dyadic symptom education to reduce incongruent perceptions of sleep disturbance in colorectal cancer dyads with a view to enhancing their quality of life. More sleep management and substantial emotional support should be provided by family caregivers and healthcare providers.

**Supplementary Information:**

The online version contains supplementary material available at https://doi.org/10.1186/s41687-026-01090-5.

## Introduction

Colorectal cancer (CRC) is one of the most prevalent forms of cancer on a global scale, and it has become a significant public health concern, occupying a leading position in terms of both incidence and mortality rates [1]. Projections indicate that the number of deaths caused by CRC in China is expected to rise from 62.9 thousand in 2016 to 67.7 thousand in 2025 [2]. A substantial symptom burden is experienced by CRC survivors, with pain, fatigue, and sleep disturbance being among the most prevalent and distressing symptoms [3]. Pain, fatigue, and sleep disturbance have been identified as the significant risk factors for the development of long-term gastrointestinal symptoms. Greater severity of pain, fatigue, and sleep disturbance was associated with worse gastrointestinal symptoms [4–6]. The severity of gastrointestinal symptoms is positively associated with poor quality of life (QOL) [7]. While effective symptom management is an integral component of QOL in CRC patients, the attainment of complete symptom relief remains elusive, resulting in a suboptimal QOL [8].

Patients suffering from CRC tend to rely on their family members as caregivers, providing them with physical, psychological, and emotional support. This reliance is further compounded by the fact that the mood of the caregiver and the nature of their intimate relationship (IR) with the patient are considered integral components of the support network [9]. This study aligned with Sternberg’s [10] definition of intimate relationships, characterized by strong feelings of closeness, connection, and physical and psychological bonding, such as parent-child, marital, sibling, or partner relationships. The role of family caregivers in the care of patients is of paramount importance, yet they are subject to considerable caregiver and emotional burdens associated with cancer treatment [11]. CRC patients and family caregivers are typically concerned about and at an increased risk of experiencing poor psychological results like negative mood (NM) [12, 13]. Recent literature has increasingly highlighted the significance of interpersonal resources in close relationships when coping with cancer. Berg and Upchurch [14] adopted a dyadic perspective, conceptualizing adjustment as a function of dyadic appraisals of the unit and dyadic coping with the demands posed by the situation. They emphasized the significance of temporal and contextual factors in this regard [14]. The development-contextual model of couples coping with illness posits that couples collaborate as an interdependent unit to evaluate, cope with, and adapt to the illness experience over time [14]. Berg [14] highlighted the importance of a dyadic insight into illness representations as one way to know the essential effect of different symptom perceptions between patients and their family caregivers. Poor adjustment is more likely when different symptom perceptions are appraised.

For instance, incongruent symptom perceptions could also be seen as disruptive, negatively impacting the caregiver burden and emotional burden within patients and caregivers [15]. The discordance in symptom perceptions between patients and their family caregivers has the potential to hinder their ability to cope with CRC, resulting in adverse consequences for family caregivers, including elevated levels of grief following bereavement [16]. Furthermore, incongruent perceptions of symptoms within the dyad have the potential to adversely impact relationship satisfaction and care outcomes [17, 18]. Congruent appraisals within the dyad are pivotal to the management of both the physical and mental health of the dyad [19]. To improve poor QOL in patient-caregiver dyads, greater involvement of family caregivers in cancer symptom management programs has been advocated, which would contribute to a better understanding of the relationships between dyadic perceptions of symptoms, mood, relationship satisfaction, and QOL [20]. However, there remains a paucity of research exploring the relationship between incongruent symptom perceptions, negative mood, intimate relationship, and QOL from a dyadic perspective.

In the extant literature, there has been a paucity of studies that have focused on the QOL of CRC dyads about their perceptions of patient symptoms, mood, and intimate relationship [21]. By examining the dyadic perceptions of symptoms, negative mood, intimate relationship, and QOL, it may be possible to gain a more profound understanding of these perceptions might associate with QOL and to determine their clinical implications for programs aimed at improving symptoms and managing negative mood and intimate relationship. The primary objective of this study was to explore the existence and identify predictors of incongruent perceptions regarding pain, fatigue, and sleep disturbance, and to examine their association with negative mood, intimate relationship and QOL within CRC dyads. We hypothesized that incongruent perceptions of pain, fatigue, and sleep disturbance would exist, and that higher levels of incongruence would be associated with worse negative mood, lower intimate relationship satisfaction, and lower QOL within these dyads.

## Methods

### Study design, patients, and procedures

This study was a cross-sectional study. A total of 271 dyads (patients and family caregivers) were recruited from the Sixth Affiliated Hospital of Sun Yat-Sen University and Nanfang Hospital in China, through convenience sampling. Inclusion and exclusion criteria for patients and caregivers, respectively, were as follows. The inclusion criteria for patients included: (1) aged 18 or older; (2) confirmed diagnosis of CRC; (3) receiving chemotherapy, with an expected lifespan of at least 6 months; (4) with at least one family caregiver; (5) informed consent to participate. Patients diagnosed with multiple primary cancer sites, as well as those with clinician-judged severe cognitive impairment or mental illness, were excluded from the study. The inclusion criteria for family caregivers were: (1) aged 18 or older; (2) living with the patient; (3) with a full understanding of the patient’s symptoms (as assessed by scales); Caregivers who had spent the most time with the patients and who were primarily responsible for their care were included, as they were considered to bear the greatest caring burden; (4) informed consent to participate. These individuals were typically the primary caregivers selected by each patient. Family caregivers who had been diagnosed with major diseases during the previous year and/or were involved in active treatment for cancer were excluded from the study. All CRC patients and family caregivers were screened by face-to-face interviews based on a screening questionnaire. Following the completion of written informed consent, the questionnaires were explained with standardized instructions, after which they were completed independently by the subjects. On the same day, patients filled out these questionnaires in their ward with a healthcare professional, and family caregivers filled out these questionnaires at the nurse station with a healthcare professional. At the time of assessment, patients and their family caregivers had not conversed about the questionnaire. The integrity of the completed questionnaire was verified at the time of the assessment, a process which took approximately 10–15 min. The study was approved by the Medical Ethics Committee of Nanfang Hospital, Southern Medical University (approval number NFEC-2020-281).

## Outcome measures

### Sociodemographic and clinical characteristics questionnaire

The demographic characteristics, sociodemographic, and clinical characteristics included age, gender, race, relationship type, relationship length, education level, religious belief, frequency of communication about symptoms, type of cancer, cancer stage of patients, chemotherapy frequency, chemotherapy cycle, time since diagnosis, operation, and ostomy.

**Patient pain severity**. The Chinese version of the Brief Pain Inventory (BPI) as developed by Cleeland [22] in 1994 was adopted to assess the perceptions of the severity of pain in the past week by patients and their family caregivers at the same time. The 3rd to the 6th items were used to rate the patient’s pain severity over the past week, based on an 11-point Likert scale ranging from 0 to 10, with 0 indicating (no pain) and 10 indicating (pain as bad as you can imagine). A higher total score indicated a higher level of pain severity. The Cronbach’s α coefficients were 0.91 and 0.93 in the patient’s perception and family caregiver’s perception, respectively, indicating good internal consistency reliability.

**Patient fatigue**. The Chinese version of the Functional Assessment Chronic Illness Therapy-Fatigue (FACIT-F), as developed by Yellen [23] in 1997 was adopted to examine the perceptions of fatigue in patients in the past week, by patients themselves and by their family caregivers at the same time concurrently. The scale addresses the perceptions of tiredness, weakness, and difficulty in conducting activities due to fatigue and has a total of 13 items, with each item rated on a 5-point Likert scale ranging from 0 to 4, with 0 indicating not at all, 1 indicating a little bit, 2 indicating somewhat, 3 indicating quite a bit, and 4 indicating very much. The total score ranges from 0 to 52, with a lower score indicating a higher level of fatigue. The Cronbach’s α coefficients were 0.93 and 0.89 in the patient’s perception and family caregiver’s perception, respectively.

**Patient sleep disturbance**. The Athens Insomnia Scale (AIS), a tool developed by Soldatos [24] in 2000, was utilized to assess the perceptions of sleep disturbance among patients and their family caregivers concurrently. The scale is composed of two parts: the first part concerns the patient’s sleep quality, while the second part concerns the interference in daily life due to sleep disturbance. The scale comprises a total of eight items, with each item rated on a 4-point Likert scale ranging from 0 to 3. A total score of < 4 would indicate no sleep disturbance, 4 to 6 would indicate suspicious insomnia, and > 6 would indicate insomnia. The total score ranged from 0 to 24, with higher scores indicating greater insomnia. The Chinese version of AIS has been demonstrated to be reliable, with a Cronbach’s α coefficient of 0.90 in the patient’s perception and the family caregiver’s perception [25, 26].

**Negative mood**. The Positive and Negative Affect Scale (PANAS) as developed by Watson [27] in 1988 was adopted to examine the perceptions of mood by patients and their family caregivers concurrently. The assessment of mood was conducted using ten items from the scale, with each item being evaluated on a 5-point Likert scale ranging from 1 to 5 (1 indicating not at all, and 5 indicating extremely). A higher score would indicate a higher level of negative mood. The Chinese version of the sub-scale has been demonstrated to possess acceptable internal consistency reliability in the patient’s perception and family caregiver’s perception, with Cronbach’s α coefficient of 0.83 and 0.84 respectively [28, 29].

**Intimate relationship**. The Relationship Assessment Scale (RAS) consisted of seven items (e.g., how well does your partner meet your needs? and how many problems are there in your relationship?), designed to evaluate satisfaction from romantic, intimate relationships [30–33]. RAS was used to measure the perceptions of intimate relationship satisfaction by patients and their family caregivers in the past week at the same time. The scale comprises a total of 7 items, with each item rated on a 5-point Likert scale ranging from 1 to 5, with 1 indicating not satisfied and 5 indicating very satisfied. Items 4 and 7 were scored inversely, the sum of scores for each item was the total score of the scale. The total score could range from 7 to 35, with a higher score indicating better intimate relationship satisfaction. The Cronbach’s α coefficient of the scale was found to be 0.82 in the patient’s perception and 0.85 in the family caregiver’s perception in the present sample. The validity of the scale was 0.85 for the patients and 0.86 for the family caregivers in the present sample.

**Quality of life**. The 12-item Short Form Health Survey (SF-12, version 1) by Kosinski in 1995 [34] was used to examine the perceptions of QOL by patients and their family caregivers in the past week at the same time. The scale is composed of two parts: physical health (PCS) and mental health (MCS). Each of these parts is further divided into eight dimensions: physiological function (PF), role physical (RP), body pain (BP), general health (GH), vitality (VT), social function (SF), role emotional (RE), and mental health (MH). Each dimensional score can be converted into a standardized total score according to a standard formula, with the total score ranging from 0 to 100. A higher total score would indicate a higher level of QOL. The Cronbach’s α coefficient of the scale was found to be 0.82 and 0.81 in the patient’s perception and family caregiver’s perception, respectively [35].

Family caregivers completed questionnaires on the perceptions of pain, fatigue, and sleep disturbance of CRC patients (but not perceptions of pain, fatigue, and sleep disturbance experienced by themselves), as they are likely to suffer from poor adjustment when they appraise different symptom perceptions. Questionnaires about the perceptions of other variables (including negative mood, intimate relationship satisfaction, and QOL) were completed by both the patients and their family caregivers, respectively.

### Statistical analysis

The primary outcome variables were subjected to descriptive statistical analysis. Paired t-tests were used to test mean differences between patients and their family caregivers on the symptom perceptions of pain, fatigue, sleep disturbance, negative mood, intimate relationship satisfaction, and QOL (PCS and MCS). Incongruent perceptions of pain, fatigue, and sleep disturbance were operationalized as the absolute difference between patient-reported and caregiver-reported scores (|patient score-caregiver score|). We used multiple linear regression to analyze the influencing factors of incongruent symptom perceptions between patients and their family caregivers. The relationships between the perceptions of patients and their family caregivers were explored through correlation analysis.

The primary objective of this study was to explore the existence of incongruent perceptions of pain, fatigue, and sleep disturbance, and their associations with negative mood, intimate relationship, and QOL in patient-caregiver dyads. Given the nested and interdependent nature of the perceptions of these outcome variables, structural equation modeling (SEM) was conducted [14]. The utilization of SEM models facilitates the examination of the interdependence of dyadic level data, such as that collected in this study, and an actor-partner mediating effect [36]. We used SEM to account for the interdependent nature of dyadic data and to test actor-partner mediation effects. The model parameters were estimated for all variables based on the bootstrap method for correcting chi-square values, using the software AMOS 21.0. The goodness-of-fit of the model was evaluated using six indices: the chi-square/degrees of freedom ratio (χ2/df), the goodness-of-fit index (GFI), the comparative fit index (CFI), the incremental fit index (IFI), the root mean square error of approximation (RMSEA), and the standardized root mean square residual (SRMR). The threshold values for adequate model fit were set at > 0.90 for GFI, > 0.95 for CFI, and < 0.08 for RMSEA and SRMR measures [37]. Given that the RAS was originally validated for intimate relationship satisfaction, we conducted a sensitivity analysis excluding non-spousal caregivers to assess the robustness of the findings.

## Results

### Characteristics of participants

A total of 293 eligible CRC patient-caregiver dyads provided consent to participate in the study. However, 22 of these dyads withdrew for personal reasons, resulting in 271 dyads who completed the study. The demographic information of patients and family caregivers is presented in Table 1. The mean age of patients was 52.0 years (SD = 11.8), while the mean age of their caregivers was 45.5 years (SD = 12.3). The majority of patients were male (62.7%), and a majority of caregivers were female (56.8%). The majority of patients (55.0%) were diagnosed with Stage Ⅳ cancer. The majority of caregivers were spouses (61.3%). The majority of patients and caregivers did not profess any religious beliefs (88.9% for patients, 84.8% for caregivers) or received college education (87.1%). A significant proportion of family caregivers (41.3%) reported having provided care for at least 30 years.

Table 2 presents the means, standard deviations, and the results of paired t-tests, which were used to identify any differences between patients and their family caregivers. Patients reported significantly higher levels of sleep disturbance compared to their family caregivers (*t* = 3.210, *p* = 0.001). Furthermore, patients reported lower levels of negative mood and poorer QOL than their family caregivers (*t*=-6.712, *p* < 0.001; *t*=-8.644, *p* < 0.001). Conversely, family caregivers reported experiencing intimacy problems to a greater extent than patients (*t*=-5.313, *p* < 0.001). The independent sample t-test was utilized to compare the difference between the symptom incongruence (SI) score and 0, thereby highlighting significant disparities in the evaluation of sleep disturbance between patients and their family caregivers. (*t*=-3.241, *p* < 0.001).

**Table 1:** Characteristics of patients and family caregivers (*N*=271dyads)

Characteristics (means ± SD)	Patients	Family caregivers
Age (years)	52.0 ± 11.8	45.5 ± 12.3
Gender (% of males)	62.7	43.2
Race (% of Han ethnicity)	96.3	98.6
Relationship (% of spouses)		61.3
College education (%)	12.9	12.9
Religious beliefs (%)	11.1	15.2
Married (%)	95.2	89.3
Frequency of communication about symptoms (%)	91.5	87.8
Colon cancer (%)	50.9	
Rectal cancer (%)	48.7	
Stage Ⅳ disease (%)	55.0	
> 30 years together (%)		41.3
Chemotherapy frequency	2.26 ± 2.14	
Chemotherapy cycle	4.24 ± 2.97	
Time since diagnosis (% of ≤ 1 year)	76.8	
Operation (%)	69.7	
Ostomy (%)	22.9	

**Table 2:** Results of differences between Patients and Family caregivers (*N*=271dyads)

Characteristics (means ± SD)	Patients	Family caregivers	t values	*p* values
Patient pain	4.69(6.30)	4.61(6.50)	1.854	0.065
Patient fatigue	32.4(11.8)	32.3(11.3)	0.038	0.963
Patient sleep disturbance	6.51(5.00)	5.64(4.40)	3.210	0.001
Negative mood	15.8(5.80)	19.1(7.40)	-6.712	<0.001
Intimate relationship	29.2(4.24)	27.6(4.40)	5.313	<0.001
SF-12 score (Physical health)	54.0(23.1)	72.7(19.0)	-8.615	<0.001
SF-12 score (Mental health)	60.4(21.5)	65.2(18.7)	-8.799	<0.001
SF-12 total score	57.2(20.6)	69.0(17.2)	-8.644	<0.001

### Multiple linear regression

Table 3 presents the results of multiple linear regression which were used to analyze the influencing factors of incongruent sleep disturbance perception. Regression analysis identified several significant predictors of incongruent sleep disturbance perceptions (*p* < 0.05), including: patient and caregiver gender and education level, patient diagnosis time, caregiver religious affiliation and marital status, and dyadic relationship type. Education level was categorized as college education or above (coded as 1) vs. not (coded as 0). Diagnosis time of the patient ≤ 1 year, religious belief, married and spouse caregivers were assessed as yes (coded as 1) or not (coded as 0). Specifically: Patient education level (B=-1.014, *p* = 0.001) and caregiver education level (B=-1.140, *p* < 0.001) demonstrated significant negative associations. Longer patient diagnosis duration (B=-1.876, *p* = 0.010) was negatively associated. Caregiver religious affiliation (B=-1.611, *p* = 0.027) and marital status (B= -3.097, *p* = 0.001) showed negative associations. Conversely: Patient gender (B = 1.361, *p* = 0.037) and caregiver gender (B = 1.803, *p* = 0.004) exhibited positive associations. Dyadic relationship type (B = 1.568, *p* = 0.043) was positively associated.

**Table 3:** Regression results of influencing factors of incongruent sleep disturbance (*N*=271dyads)

Constant	B	S.E.	t values	*P* values	95%CI
	10.114	4.735	2.136	0.034	0.788～19.440
**Patients**					
Age	0.023	0.032	0.735	0.463	-0.039～0.085
Gender	1.361	0.650	2.094	0.037	0.081～2.641
Race	2.002	1.438	1.393	0.165	-0.829～4.833
Education	-1.014	0.314	-3.229	0.001	-1.632～-0.395
Religious beliefs	1.066	0.919	1.161	0.247	-0.743～2.875
Marital status	-0.292	1.200	-0.243	0.808	-2.656～2.072
Cancer type	-0.934	0.563	-1.660	0.098	-2.043～0.174
Cancer stage	-0.334	0.344	-0.971	0.332	-1.012～0.344
Chemotherapy frequency	0.176	0.142	1.239	0.216	-0.104～0.455
Chemotherapy cycle	0.119	0.099	1.204	0.230	-0.076～0.314
Time since diagnosis	-1.876	0.726	-2.585	0.010	-3.304～-0.447
Operation	-0.356	0.646	-0.550	0.583	-1.629～-0.917
Ostomy	1.131	0.680	1.664	0.097	-0.207～2.469
**Family caregivers**					
Age	0.014	0.033	0.433	0.666	-0.051～0.080
Gender	1.803	0.624	2.887	0.004	0.573～3.032
Race	-0.320	0.335	-0.953	0.342	-0.980～0.341
Education	-1.140	0.318	-3.588	<0.001	-1.766～-0.514
Religious beliefs	-1.611	0.725	-2.222	0.027	-3.039～-0.183
Marital status	-3.097	0.893	-3.466	0.001	-4.856～-1.337
Relationship type	1.568	0.771	2.035	0.043	0.050～3.086
Relationship Length	-0.864	0.727	-1.188	0.236	-2.295～0.568

### Correlations analysis

Table 4 shows the correlations among the primary study variables for colorectal cancer (CRC) patients and their family caregivers. For CRC patients, incongruent sleep disturbance (ISD) demonstrated a positive correlation with their negative mood (*r* = 0.159, *p* < 0.01) and a negative correlation with their own QOL (*r*=-0.015, *p* < 0.05). Contrary to expectations, no correlation was found between incongruent sleep disturbance and the intimate relationship satisfaction of CRC patients. Conversely, negative mood demonstrated a negative correlation with CRC patients’ intimate relationship satisfaction (*r*=-0.179, *p* < 0.01) and QOL (*r*=-0.247, *p* < 0.001). No correlation was observed between negative mood and the intimate relationship satisfaction or QOL of caregivers. Conversely, intimate relationship satisfaction was found to be positively correlated with both CRC patients (*r* = 0.155, *p* < 0.05) and caregivers’ QOL (*r* = 0.126, *p* < 0.05). For caregivers, incongruent sleep disturbance demonstrated a positive correlation with intimate relationship satisfaction(*r* = 0.148, *p* < 0.05) and QOL (*r* = 0.121, *p* < 0.05), and a negative correlation with the negative mood (*r*=-0.209, *p* < 0.01) of caregivers. Conversely, negative mood demonstrated a negative correlation with their own intimate relationship satisfaction (*r*=-0.166, *p* < 0.01) and QOL (*r*=-0.337, *p* < 0.001). No correlation was observed between CRC patients’ intimate relationship satisfaction and QOL. Conversely, a positive correlation was observed between intimate relationship satisfaction and QOL (*r* = 0.371, *p* < 0.001). The sensitivity analysis excluding non-spousal caregivers presented similar results to the primary analysis in terms of the direction and significance of the main associations.

**Table 4:** Correlations between incongruent sleep disturbance, negative mood, intimate relationship and QOL among patients and caregivers (*N* = 271 dyads)

	ISD	NM-*P*	NM-FC	IR-*P*	IR-FC	QOL-*P*	QOL-FC
ISD	1	0.159**	-0.209**	-0.037	0.148*	-0.015*	0.121*
NM-P	0.159**	1	0.248**	-0.179**	-0.063	-0.247***	-0.082
NM-FC	-0.209**	0.248***	1	-0.106	-0.166**	-0.049	0.126*
IR-P	-0.037	-0.179**	-0.106	1	0.348**	0.155*	-0.337***
IR-FC	0.148*	-0.063	-0.166**	0.348**	1	0.059	0.126*
QOL-P	-0.015*	-0.247***	-0.049	0.155*	0.059	1	0.371***
QOL-FC	0.121*	-0.082	-0.337***	0.126*	0.371***	0.067	1

Note. *p < 0.05,**p < 0.01,***p < 0.001. ISD=incongruent sleep disturbance; P=patient; FC= family caregiver; NM=negative mood; IR=intimate relationship; QOL=quality of life

### Mediation analysis with Structural equation modeling (SEM)

**Fig. 1:**
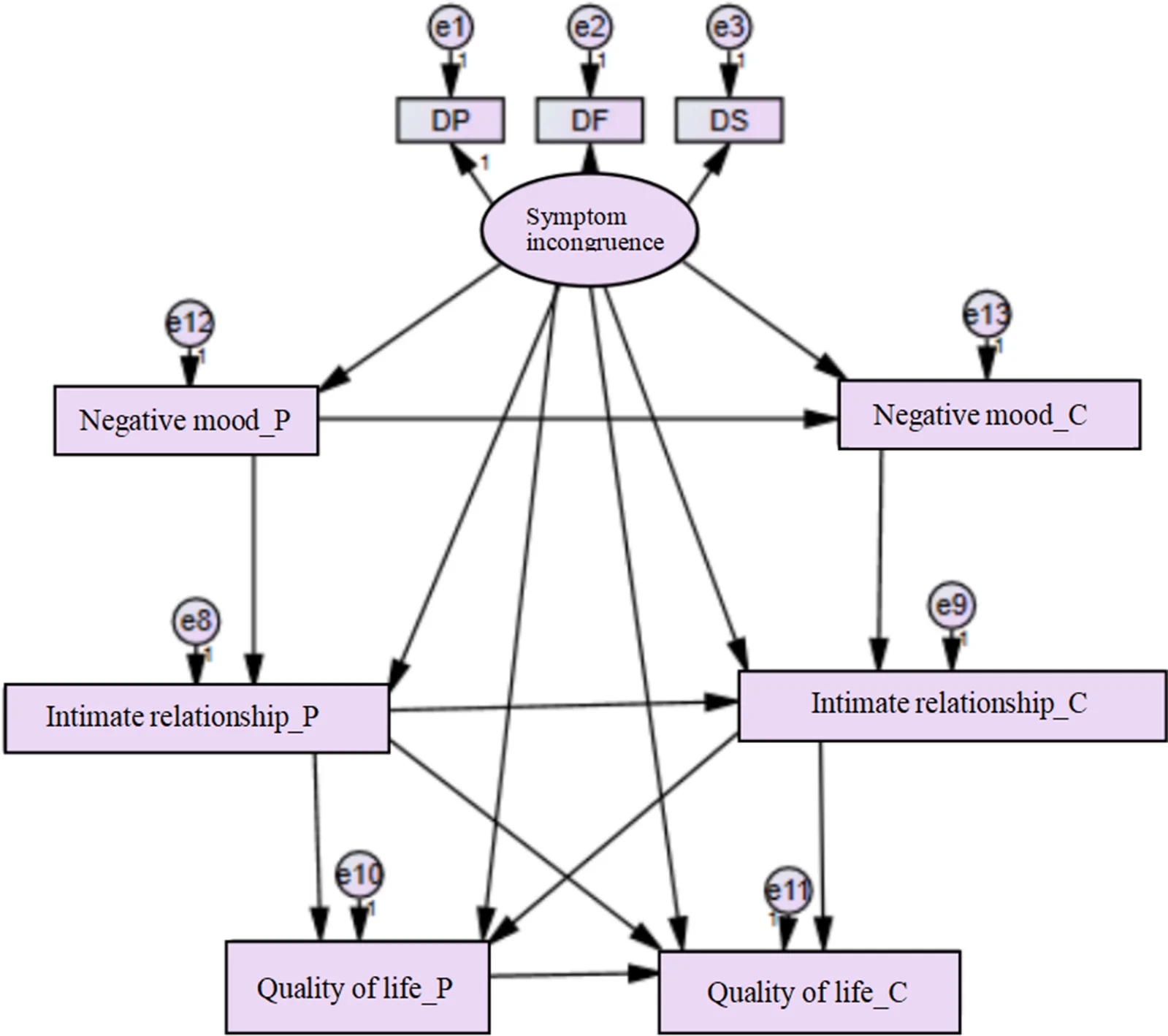
Structural equation model of dyadic symptom incongruence, negative mood, intimate relationship, and quality of life. Note: DP=different pain; DF=different fatigue; DS=different sleep disturbance; P=patient; C=caregiver

Potential confounding variables (e.g., gender, relationship type, cancer stage, chemotherapy frequency, chemotherapy cycle, time since diagnosis, operation, ostomy) were considered for inclusion as control variables in the SEM analysis. The proposed SEM is illustrated graphically in Fig. 1, based on the available data.

Pathway analysis demonstrates the actor effect and partner effect of symptom incongruence, negative mood, intimate relationship and QOL. As demonstrated, symptom incongruence of different sleep disturbance (DS) through negative mood and intimate relationship may exert a direct or indirect influence on QOL as actor effects and/or partner effects in the two models, respectively. The findings reveal a significantly direct effect of symptom incongruence of sleep disturbance on the QOL of patients and their family caregivers, albeit in opposite directions (B=-0.238, *p* < 0.001; B = 0.358, *p* < 0.001, respectively). Incremental fit measures, such as the χ2/df, GFI, IFI, CFI, RMSEA, and SRMR, can be utilized to ascertain the extent to which the SEM aligns with the data. In the present study, the estimated model fit parameters were as follows: χ2/df = 1.570, GFI = 0.967, CFI = 0.957, IFI = 0.922, RMSEA = 0.036, SRMR = 0.053. The SEM model demonstrated a high degree of compatibility with the data, as illustrated in Table 5.

These SEM results suggested that indirect effects were mediated by negative mood and intimate relationship, as illustrated in Fig. 1. The pathways from symptom incongruence to negative mood to intimate relationship to QOL of patients (QOL_P) and QOL of caregivers (QOL_C) and those from QOL of caregivers to QOL of patients were found to be significantly different (*p* < 0.001). The results of the pathway analysis are summarised in Table 6. The findings revealed the presence of an actor effect and a partner effect between symptom incongruence and negative mood in CRC patients and caregivers. Additionally, a partner effect was observed between symptom incongruence and intimate relationship in CRC patients and caregivers, but no actor effect was detected between symptom incongruence and intimate relationship. Conversely, the results indicated an actor effect and a partner effect between symptom incongruence and QOL in CRC patients and caregivers. In conclusion, the findings substantiate the existence of an actor-partner mediating effect pertaining to symptom incongruence, negative mood, intimate relationship, and QOL.

**Table 5:** Overall goodness of fit analysis table for the SEM model

Parameters	Ideal standard	General standard	Result	Conclusion
CMIN/DF	1–3	The smaller the better	1.570	good
RMSEA	< 0.08	< 0.1	0.036	good
SRMR	< 0.08	< 0.1	0.053	good
GFI	> 0.90	> 0.8	0.967	good
CFI	> 0.95	> 0.8	0.957	good
IFI	> 0.90	> 0.8	0.922	good

**Table 6:** Standard pathway analysis coefficients for the proposed SEM model (Fig. 1)

Pathway	B	S.E.	C.*R*	*P* values
NM_P←SI	0.188	0.291	2.098	0.036
IR_P←SI	0.010	0.189	0.126	0.900
NM_C←SI	-0.422	0.482	-3.577	< 0.001
NM_C←NM_P	0.314	0.076	5.201	< 0.001
IR_P←NM_P	-0.174	0.045	-2.817	0.005
IR_C←SI	0.073	0.207	0.854	0.393
IR_C←IR_P	0.340	0.059	5.993	< 0.001
IR_C←NM_C	-0.108	0.038	-1.672	0.095
QOL_P←SI	-0.238	1.083	-2.505	0.012
QOL_P←IR_P	-0.018	0.275	-0.315	0.752
QOL_P←IR_C	0.021	0.266	0.372	0.710
QOL_C←SI	0.358	0.991	3.433	< 0.001
QOL_C←IR_P	0.065	0.226	1.158	0.247
QOL_C←IR_C	0.321	0.222	5.644	< 0.001
QOL_C←QOL_P	0.370	0.046	6.629	< 0.001

Note. SI=symptom incongruence; P=patient; C=caregiver; NM=negative mood; IR=intimate relationship; QOL=quality of life

## Discussion

Initially, no substantial disparities were observed in patient pain and fatigue ratings, as perceived by patients and family caregivers. However, patient sleep disturbances were reportedly underestimated by family caregivers in our study. And this is inconsistent with Lyons’s conclusions [16]. Secondly, regression analysis identified several significant predictors of incongruent sleep disturbance perceptions, including: patient and caregiver gender and education level, time since diagnosis, caregiver religious affiliation and marital status, and dyadic relationship type. Thirdly, family caregivers exhibited poorer negative mood, intimate relationship satisfaction, and better QOL in comparison to patients. Then, the dyadic SEM model indicated that incongruent perceptions of sleep disturbance had associations with the QOL in CRC patient-family caregiver dyads, and negative mood and intimate relationship could mediate the relationship between this incongruent perception and the QOL.

Despite the recent focus among researchers on the symptom agreement or incongruence between patients and their caregivers, there is a paucity of studies on sleep disturbance, the prevalence of which was 58.5% in cancer patients [38]. Consistent with the findings of Poort [39], the perceptions of pain and fatigue in patients by family caregivers were found to be similar to those of the patients themselves. In line with earlier research on dyadic symptom appraisal, where family caregivers assessed patient symptoms more negatively, our study revealed that family caregivers underestimated patients’ sleep disturbances [40, 41]. One potential explanation for this phenomenon is that family caregivers are more likely to observe the patient’s pain and fatigue than sleep disturbances, resulting in a low level of incongruence between the patient and the caregiver regarding these symptoms [40, 42]. In the present study, 38.7% of family caregivers were non-spouses, non-spouse caregivers often sleep in separate beds with patients, lacking the opportunity to closely observe the patients’ sleep related behaviors such as turning over, groaning, waking up at night, breathing changes at night, which may have resulted in an inaccurate observation of sleep disturbances. Further research is required to explore the patterns of incongruence between patients and family caregivers and to generalize these findings across different cultures over time.

The present study found that family caregivers exhibited poorer negative mood and intimate relationship satisfaction compared to CRC patients. These findings contradict those of previous studies, which suggested that patients experience worse negative mood and relationship satisfaction [43, 44]. Potential explanations for this discrepancy may include the following. First, a majority of patients in this study (55%) presented with Stage IV disease, a context in which caregivers commonly experience anticipatory grief and fear of impending loss. These psychological stressors frequently contribute to substantial emotional burdens and negative mood states [45]. Second, due to the severity of patients’ conditions, caregivers often provide continuous nursing care. This frequently necessitates career interruptions through resignation or extended leave, contributing to substantial physical strain and social role dislocation. Concurrently, care-related expenses (including medical costs and supportive care supplies) generate considerable financial strain. Consequently, family caregivers may encounter heightened negative mood. The study’s findings were consistent with those reported by Roberson [46] based on caregiver reports of intimate relationship quality. The current study observed a significant proportion of female spouse caregivers, a demographic that may be predisposed to perceiving diminished marital relationship quality [47]. Another potential explanation is that 74.9% of patients argued that they often communicated cancer-related symptoms with their family caregivers. And the more frequently patients communicated with family caregivers, the higher level of relationship they may reported [48]. Further, family caregivers reported better QOL than patients no matter physical health and mental health. This finding is parallel with Li’s [49] study. CRC patients experienced some side effects of surgery like sexual dysfunction. It was known that sexual dysfunction may greatly damage CRC patient’s QOL [50]. So CRC patients reported worse QOL than family caregivers.

Caregivers often overestimate the severity of patients’ symptoms, and the accuracy decreases over time [51]. Identifying factors influencing the accuracy of symptom assessment within CRC caregiver-patient dyads is critically important. Our study revealed that female caregiver-patient dyads demonstrated significantly higher symptom assessment concordance compared to male dyads. This finding aligns with prior research [42, 52]. Strongly influenced by patriarchal cultural norms and familial values, Chinese males are often socialized towards independence, emotional resilience, and occupying provider/protector roles within the family unit [53]. To enhance the symptom perception concordance between male caregivers and patients, we recommend that male caregivers should participate in daily morning round, unify understanding and recognition of symptoms with patients, and clarify their caregiving responsibilities. For male caregivers, they need to communicate more with patients and pay attention to their discomfort, and regularly hold family meetings to discuss symptom perception concordance. Medical workers also should take the initiative to care for male caregivers and acknowledge their efforts.

Caregiver relationship type and marital status emerged as significant predictors of assessment concordance, corroborating Chen’s [54] findings. Within spousal caregiving relationships, patients may experience heightened emotional support and affection, potentially facilitating greater openness in communication and symptom disclosure [55]. Given that the majority of patients and caregivers in this study were married cohabitants, spousal caregivers possess greater opportunity for direct nocturnal observation of patients.

Dyads wherein both members received college-level education demonstrated superior assessment accuracy relative to those with lower educational attainment, consistent with Silveira’s [51] findings. While only 12.9% of patients and caregivers in this study held college degrees, shared educational background appears to foster a mutual understanding of the patient’s symptom experience, thereby contributing to concordant assessment. Higher educational attainment typically correlates with improved health literacy and enhanced capacity to comprehend symptom-related educational materials. Consequently, caregivers with advanced education are more likely to proactively acquire knowledge pertinent to symptom recognition and management.

Our analysis also underscored the need for heightened clinical attention toward dyads characterized by shorter time since patient diagnosis and non-religious caregivers. Patients with a shorter diagnostic interval may present with less overt or nascent symptoms, complicating detection. Simultaneously, caregivers in these dyads have had less time to develop proficiency in symptom recognition, potentially impairing their ability to accurately perceive the patient’s experience. Furthermore, religiosity was positively associated with enhanced compassionate capacity and interpersonal attunement [55]. Caregivers exhibiting religiosity demonstrated a heightened ability to comprehend and empathize with the patient’s subjective experiences.

This study constituted a rare attempt to explore the differences in dyadic symptom perceptions and thier associations with QOL in CRC patient-family caregiver dyads. The findings, derived from Structural Equation Modelling analysis, offer novel insights. The current study revealed a significant mediating role of negative mood and intimate relationship, which may reveal the underlying mechanism of how incongruent sleep disturbance indirectly influences QOL. A body of research has indicated that cancer patients and their family caregivers are interdependent, with both dyad members exhibiting a partner effect [56]. For instance, the patient’s QOL was found to be related to the caregiver’s mood over time [57]. These findings are consistent with the developmental-contextual framework, which supports the important effects of shared symptom appraisal in the chronic illness context, such as cancer [14]. The results of the current study provide preliminary evidence that incongruent perceptions of sleep disturbance have a significant association with the QOL of CRC patients and their family caregivers. The findings of this study demonstrated that QOL of patients and their family caregivers was associated with dyadic symptom appraisal, suggesting that the perception of sleep disturbance incongruence exerted a significant direct association with the QOL of patients and caregivers, consistent with previous studies [58–63]. Of particular note was the finding that incongruent perceptions of sleep disturbance could also play a role in dyadic QOL, mediating negative mood and intimate relationship. Patients may be more likely to perceive a lack of support from their partners when family caregivers underestimate their symptoms [64]. This can result in poorer negative mood among cancer patients [65]. Research has demonstrated a correlation between mood and intimate relationship satisfaction among cancer patients [66, 67]. Spillover has been demonstrated to occur from caregivers to patients and from patients to caregivers [57]. Furthermore, the presence of negative moods and intimate relationships has been identified as a predictor of QOL. Consequently, the present study hypothesizes that incongruent sleep disturbance exerts a indirect association with the QOL through negative mood and intimate relationship. In light of these findings, we propose that clinicians develop interventions to strengthen concordant sleep assessment to enhance the QOL of CRC patients and family caregivers. Furthermore, interventions aimed at enhancing positive mood and intimacy can be considered as a means to improve the QOL in both CRC patients and family caregivers.

### Study limitations

The present study was subject to several limitations. Although, sensitivity analyses excluding non-spousal caregivers yielded consistent results, supporting the robustness of our main findings. This study included family caregivers who were not spouses, the applicability of the RAS to non-spousal caregiver relationships remains a consideration for future research. Future studies should prioritize the recruitment of spousal caregivers. Furthermore, methodological constraints related to time and venue accessibility limit, the cross-sectional design and convenience sampling of the study may have introduced biases, precluding the analysis of changes in incongruent perceptions and relationships among the outcome variables over time. We acknowledge that the operationalization of incongruent symptom perception as an absolute difference score, while common, may not fully capture the complexity of dyadic congruence. Response surface analysis (RSA) has been proposed as a more nuanced approach for examining congruence effects in dyadic data [68]. Future studies may consider applying RSA to further elucidate the patterns of symptom perception congruence. It is therefore recommended that future studies include longitudinal and larger samples of dyads with life-threatening diseases such as cancer and cardiovascular disease. Third, it is noteworthy that patients participating in the present study reported a lower level of pain (M = 4.69), which may have influenced incongruent perceptions between patients and family caregivers. This study’s sampling approach likely introduced selection bias, as patients without their primary caregivers present were systematically excluded. Consequently, the final cohort was skewed toward majorities of male patients and late-stage disease representation (55% Stage IV). Finally, the presence of other cancer-related symptoms may limit the generalisability of the findings regarding dyadic symptom incongruence. For instance, symptoms such as nausea and vomiting were not measured in the present study.

## Conclusion

In conclusion, dyadic studies provide valuable insight into the processes at work for those facing the pressure of CRC. The present study provides significant guidance for cancer patients and their family caregivers, to enhance the management of symptoms and the QOL in a clinical context. Concomitantly, we emphasise the multifaceted role of negative mood and the intricacies of the intimate relationship within the dyad in a life-threatening context. These observed interdependencies indicate that dyadic interventions targeting sleep incongruence, negative mood, and intimate relationship may enhance QOL in CRC patient-caregiver dyads. Consequently, healthcare providers should reinforce patient-caregiver dyadic symptom education and structured open communication such as discussing symptom incongruence with healthcare providers to improve concordance. More sleep management and substantial emotional support should be provided by family caregivers and healthcare providers for CRC patients.

## Supplementary Information

Below is the link to the electronic supplementary material.

Supplementary Material 1

## Data Availability

The datasets used and analyzed during the current study are available from the corresponding author on reasonable request.
