# Supplementary material for: Incongruent symptom perceptions and its associations with negative mood, intimate relationship and quality of life in colorectal cancer dyads: a dyadic analysis
Source: J Patient Rep Outcomes. 2026 Jun 6;10:164. doi: 10.1186/s41687-026-01090-5 (PMC13545201; doi:10.1186/s41687-026-01090-5)
Supplement: Supplementary file 1 [file 41687_2026_1090_MOESM1_ESM.docx]

**Supplementary Table S1** Sensitivity analysis excluding non-spousal caregivers (N=166 dyads)

| **Analysis** | **Variable** | **Primary Analysis**  **(N=271)** | **Sensitivity Analysis**  **(N=166)** | **Consistency** |
| --- | --- | --- | --- | --- |
| **Paired *t*-test** |  | ***t* values** | ***t* values** |  |
|  | Patient sleep disturbance | 3.210*** | 8.467*** | Consistent |
|  | Negative mood | -6.712*** | -6.543*** | Consistent |
|  | Intimate relationship | 5.313*** | 5.307*** | Consistent |
|  | SF-12 score  (Physical health) | -8.615*** | -7.985*** | Consistent |
|  | SF-12 score  (Mental health) | -8.799*** | -7.044*** | Consistent |
|  | SF-12 total score | -8.644*** | -5.145*** | Consistent |
| **Multiple linear regression** |  | **B (95%CI)** | **B (95%CI)** |  |
|  | Patient gender  (ref: female) | 1.361*  (0.081, 2.641) | 1.402*  (0.112, 2.692) | Consistent |
|  | Caregiver gender  (ref: female) | 1.803**  (0.573, 3.032) | 1.756**  (0.521, 2.991) | Consistent |
|  | Patient education  (college vs. not) | -1.014**  (-1.632, -0.395) | -1.085**  (-1.712, -0.458) | Consistent |
|  | Caregiver education (college vs. not) | -1.140***  (-1.766, -0.514) | -1.203***  (-1.835, -0.571) | Consistent |
|  | Patient time since diagnosis  (≤1 year vs. >1 year) | -1.876*  (-3.304, -0.447) | -2.155*  (-3.281, -0.142) | Consistent |
|  | Caregiver religious belief (yes vs. no) | -1.611*  (-3.039, -0.183) | -2.322*  (-4.182, -0.337) | Consistent |
|  | Caregiver marital status (married vs. not) | -3.097**  (-4.856, -1.337) | -3.201**  (-4.972, -1.430) | Consistent |
|  | Relationship type  (spouse vs. other) | 1.568*  (0.050, 3.086) | — | — |
| **SEM** |  | **B (SE)** | **B (SE)** |  |
|  | QOL_P←SI | -0.238** (1.083) | -0.251** (1.091) | Consistent |
|  | QOL_C←SI | 0.358*** (0.991) | 0.341*** (0.985) | Consistent |
|  | NM_P←SI | 0.188* (0.291) | 0.195* (0.288) | Consistent |
|  | NM_C←SI | -0.422*** (0.482) | -0.398*** (0.476) | Consistent |
|  | IR_P←NM_P | -0.174***(0.045) | -0.169***(0.044) | Consistent |
|  | IR_C←IR_P | 0.340***(0.059) | 0.335*** (0.058) | Consistent |
|  | QOL_C←IR_C | 0.321***(0.222) | 0.318*** (0.220) | Consistent |
|  | QOL_P←QOL_C | 0.370*** (0.046) | 0.382*** (0.045) | Consistent |

Note. SI = symptom incongruence (sleep disturbance); P=patient; C=caregiver; NM=negative mood; QOL=quality of life. Relationship type was omitted from the sensitivity analysis because all caregivers in this subsample were spouses.

*p < 0.05, **p < 0.01, ***p < 0.001.
